# Gonadotropin-releasing hormone receptor activates GTPase RhoA and inhibits cell invasion in the breast cancer cell line MDA-MB-231

**DOI:** 10.1186/1471-2407-12-550

**Published:** 2012-11-23

**Authors:** Arturo Aguilar-Rojas, Maira Huerta-Reyes, Guadalupe Maya-Núñez, Fabián Arechavaleta-Velásco, P Michael Conn, Alfredo Ulloa-Aguirre, Jesús Valdés

**Affiliations:** 1 Centro de Investigación Biomédica del Sur (CIBIS), Instituto Mexicano del Seguro Social (IMSS), Argentina No. 1, Col. Centro, 62790, Xochitepec, Morelos, Mexico; 2 Research Unit in Reproductive Medicine, Unidad Médica de Alta Especialidad-Hospital de Ginecobstetricia No. 4 “Luis Castelazo Ayala” IMSS, Mexico, DF, Mexico; 3 Oregon National Primate Research Center, Oregon Health Sciences University, Beaverton, OR, USA; 4 Division of Reproductive Health, Research Center in Population Health, National Institute of Public Health, Cuernavaca, Morelos, Mexico; 5 Departament of Biochemistry, Centro de Investigación y de Estudios Avanzados (CINVESTAV), Apartado Postal 14-740, Mexico, DF, 07000, Mexico

**Keywords:** Gonadotropin-releasing hormone receptor (GnRHR), Gonadotropin-releasing hormone (GnRH), RhoA GTPase, Cell migration, Cell adhesion, Buserelin

## Abstract

**Background:**

Gonadotropin-releasing hormone (GnRH) and its receptor (GnRHR) are both expressed by a number of malignant tumors, including those of the breast. In the latter, both behave as potent inhibitors of invasion. Nevertheless, the signaling pathways whereby the activated GnRH/GnRHR system exerts this effect have not been clearly established. In this study, we provide experimental evidence that describes components of the mechanism(s) whereby GnRH inhibits breast cancer cell invasion.

**Methods:**

Actin polymerization and substrate adhesion was measured in the highly invasive cell line, MDA-MB-231 transiently expressing the wild-type or mutant DesK191 GnRHR by fluorometry, flow cytometric analysis, and confocal microscopy, in the absence or presence of GnRH agonist. The effect of RhoA-GTP on stress fiber formation and focal adhesion assembly was measured in MDA-MB-231 cells co-expressing the GnRHRs and the GAP domain of human p190Rho GAP-A or the dominant negative mutant GAP-Y1284D. Cell invasion was determined by the transwell migration assay.

**Results:**

Agonist-stimulated activation of the wild-type GnRHR and the highly plasma membrane expressed mutant GnRHR-DesK191 transiently transfected to MDA-MB-231 cells, favored F-actin polymerization and substrate adhesion. Confocal imaging allowed detection of an association between F-actin levels and the increase in stress fibers promoted by exposure to GnRH. Pull-down assays showed that the effects observed on actin cytoskeleton resulted from GnRH-stimulated activation of RhoA GTPase. Activation of this small G protein favored the marked increase in both cell adhesion to Collagen-I and number of focal adhesion complexes leading to inhibition of the invasion capacity of MDA-MB-231 cells as disclosed by assays in Transwell Chambers.

**Conclusions:**

We here show that GnRH inhibits invasion of highly invasive breast cancer-derived MDA-MB-231 cells. This effect is mediated through an increase in substrate adhesion promoted by activation of RhoA GTPase and formation of stress fibers and focal adhesions. These observations offer new insights into the molecular mechanisms whereby activation of overexpressed GnRHRs affects cell invasion potential of this malignant cell line, and provide opportunities for designing mechanism-based adjuvant therapies for breast cancer.

## Background

Breast cancer is the main cause of death from cancer in women. In terms of number of new cases, this malignancy represents the third most frequent cancer and the ratio of mortality to incidence is about 61% [1]. Chemotherapy is central in the treatment of breast cancer, however it is well known that antineoplastic agents may cause serious adverse and toxic effects [2]. Although malignant breast tumors can be responsive to initial chemotherapy, the development of intrinsic or acquired multidrug resistance limits malignant tumor cells treatments and restricts subsequent responses to therapy [2,3]. Development and growth of metastases at distant sites are the principal cause of death among breast cancer patients, being responsible for approximately 90% of deaths from this malignant disease [4,5]; further, in metastatic tumors, the response rates to first line chemotherapies, either by single or combined drugs, range from 30-70% with remission periods following treatment of only 7–10 months [3]. Therefore, the development of alternative therapies to prevent or ameliorate the fatal course of this disease is essential.

The metastatic process comprises an ordered series of events in which the acquisition of a motile and invasive phenotype to penetrate the extracellular matrix (ECM) is one of the earliest steps and a key determinant of the invasive potential of tumor cells [6]. During cell migration, the so-called focal adhesion complex (FA) serves as a point of control for cell migratory potential by regulating the continuous formation and turnover of cell substratum contacts as well as actin polymerization. The regulation of actin cytoskeleton during cell locomotion and adhesion is performed by small G proteins from the Rho family, which comprises several members, including RhoA, Rac1, and Cdc42 [7]. RhoA is responsible for the development of stress fibers and focal adhesion assembly [8]. Although the specific mechanisms that control the assembly of the FA and cell substrate-adhesion factors are not well understood, the importance of RhoA in this process has been demonstrated by *in vitro* studies. For example, in cultured cells low levels of activated-RhoA have been found to be associated with a high migration phenotype [9,10] whereas, in contrast, high RhoA activity has been linked to poor migration ability by high substrate adhesion [11-13]. Thus, it appears that RhoA is a key regulator of cell adhesion and motility in cancer cells.

Gonadotropin-releasing hormone (GnRH), a decapeptide synthesized in the hypothalamus, and its receptor, the gonadotropin-releasing hormone receptor (GnRHR), a G protein-coupled receptor located in the membrane of the gonadotrophs of the anterior pituitary [14], are key regulators of reproductive function. However, it has been found that the GnRHR is not exclusively expressed in the anterior pituitary gland but also in other reproductive tissues such as the breast, endometrium, ovary, and prostate as well as in tumors derived from these tissues, where it probably regulates cell proliferation and tumor invasiveness [15-18]. In fact, GnRH and some of its agonists have shown to be effective in controlling tumor growth and invasiveness in *in vitro* and *in vivo* systems [19-21]. Further, several studies have shown that the ability of the GnRH/GnRHR system to reduce cell tumor invasion and metastatic potential are associated with up regulation of actin cytoskeleton remodeling, mainly through the activation of Rac1 [22,23] as well as by influencing the activity of cell-cell adhesion molecules and/or the regulation of cell substrate attachment-associated proteins [24,25]. These observations have provided new insights into opportunities for adjuvant therapies based on disruption of these processes.

Approximately 50-60% of breast cancer tumors as well as several breast cancer-derived cell lines express specific binding sites for GnRH [26,27]. The role of GnRH and GnRH agonists (GnRHa) to inhibit growth of breast cancer cells has been demonstrated in both *in vitro*[18] and *in vivo* models [15,16,19]. Likewise, the ability of GnRH and GnRHa to reduce the migratory potential of these cells has also been established [20,21]. Nevertheless, at this point much less is known about the molecular mechanisms subserving the effects of the GnRH/GnRHR system to inhibit breast cancer cells migration. A key point in this process might be the regulation of the cytoskeleton and extracellular matrix (ECM)-adhesion.

In the present study, we analyzed the molecular mechanisms employed by the human GnRHR to regulate cell motility in the highly invasive breast cancer cell line MDA-MB-231. We found that GnRHR activation by the GnRHa, Buserelin, affected several cellular markers of locomotion, including actin organization and polymerization as well as active RhoA-GTP levels. The cellular modifications observed correlated with high levels of cell adhesion and FA assembly, and inhibition of trans-well invasion.

## Methods

### Cell culture

The highly invasive breast cancer cell line, MDA-MB-231 (MDA) [28] was obtained from the American Type Culture Collection (ATCC, Manassas, VA, USA). The MDA cells were cultured in Leibovitz’s medium supplemented with antibiotics and 10% fetal calf serum (FCS) (Invitrogen, Carlsbad CA, USA) in a humidified chamber at 37°C and 5% CO_2_. The breast cancer line MCF-7 (ATCC), was cultured in Dulbecco’s modified Eagle’s medium (DMEM) (Invitrogen) supplemented with 10% FCS and antibiotics at 37°C and 5% CO_2_ in a humidified atmosphere.

### Constructions

Wild-type (WT) GnRHR (GeneBank access number L07949; [29]) and mutant GnRHR lacking lysine at position 191 (at the extracellular loop 2) (GnRHR-DesK191) cDNAs, cloned in the expression vector pcDNA3.1 (Invitrogen) at Kpn1 and Xba1 sites (New England BioLabs, Ipswich MA, USA) were synthesized as described previously [30]. As previously shown [31], the GnRHR-DesK191 is expressed at higher levels compared to the WT receptor. The coding cDNA region of the human guanine activating protein domain (GAP; amino acid residues 1248 to 1431) of the Rho-activating protein, p190Rho GAP-A (GeneBank access number AF159851; [32]) was isolated from total MCF-7 cells RNA by RT-PCR, and cloned into the pcDNA3.1 vector at the restriction site Xho1 (New England BioLabs). The dominant negative mutant of the GAP domain (GAP-Y1284D) [33], was constructed employing the QuickChange site-directed mutagenesis kit (Stratagene, La Jolla, CA, USA); the mutagenic oligonucleotide primers (Invitrogen) were designed according to the sequence of the GAP domain mentioned above. The fidelity of all constructions was verified by dye terminator cycle sequencing (Perkin Elmer, Foster City CA, USA).

### Transient transfection of MDA-MB231 cells

Wild-type and modified cDNA constructions were transiently expressed in MDA cells. Transfections (800 ng DNA/well) were performed employing the FuGENE HD transfection reagent (Roche Applied Science, Sandhofer, Mannheim, Germany) following the manufacturer’s protocol. Briefly, MDA cells were trypsinized and ~250,000 cells/well were plated in 12-well culture plates (Costar, Cambridge, MA, USA). For co-transfections, cells were transfected with WT GnRHR and GAP domain cDNAs (GAP cells) or WT GnRHR and GAP-Y1284D domain (GAP-Y1284D cells) cDNAs at a 1:1 ratio. Experiments were performed 24 hours after transfection. Cells transfected with empty pcDNA3.1 vector were employed as negative controls.

### Radioligand binding assays

Radioligand binding assays were performed as previously described [34]. Briefly, 100,000 cells per well were plated in 24-well plates (Costar) and transfected as described above. Twenty-four hours after start the transfection, cells were washed twice with Lebovitz medium and 0.1% BSA (Sigma, St. Louis MO, USA), and kept in FCS-free growth media for 18 hours. Thereafter, cells were washed twice and incubated at room temperature for 90 minutes in the presence or absence of excess (10 μM) unlabeled Buserelin (Sigma) plus ^125^I]-Buserelin (specific activity, 700 mCi/mg). After the incubation, the medium was removed and the cells were washed twice with ice-cold PBS. Cells were then solubilized in 0.2 M NaOH/0.1% SDS and counted.

### Measurement of inositol phosphate (IP) production

Inositol phosphates (IP) production was measured in cells cultured in inositol phosphate-free medium and preloaded with 4 μCi/ml ^3^H]-myo-inositol (New England Nuclear, Boston MA, USA) for 18 hours at 37°C, as previously described [31,35]. Transfected cells (50,000 cells/well) were exposed to Buserelin (10-^11^ to 10^-7^ M) for 2 hours and then washed twice with inositol-free medium supplemented with 5 mM LiCl. Quantification of IP was determined by Dowex anion exchange chromatography and liquid scintillation spectroscopy.

### Measurement of F-actin

The amount of actin polymerized (F-actin) in adherent cells stimulated with Buserelin was determined by fluorometry [36] in transfected cells (250,000 cells/well) stimulated with 10^-7^M Buserelin for 24 hours. Cells were then fixed with 3.7% formaldehyde (Sigma) in PBS for 10 minutes, and permeabilized with 0.1% Triton X-100 (Sigma) in PBS for 1 minute. F-actin was stained by incubating with 0.165 mM rhodamine-conjugated phalloidin (Molecular Probes, Eugene OR, USA) during 20 minutes in the dark at room temperature. Rhodamine bound to F-actin was removed with methanol and read in a Fluroskan Ascent Microplate Fluorometer (Thermo Scientific, USA) at 554 nm for excitation and 573 nm for emission. To determine the relative amount of rhodamine bound to F-actin per cell, five randomized fields per well were counted after methanol extraction [37]. The relative F-actin content was expressed as the amount of rhodamine-phalloidin per cell in Buserelin-stimulated samples divided by the amount of rhodamine-phalloidin per cell in control samples [38].

The amount of F-actin in suspended, GnRHa-stimulated cells was determined by flow cytometric analysis [36]. Briefly, transfected cells in suspension (50,000 cells/tube) were incubated in the absence or presence of 10^-7^ M Buserelin for 2 hours at 37°C. Cell suspensions were then fixed with 3.5% formaldehyde and quenched in 0.1 M glycine for 30 minutes. After permeabilizing with 0.2% Triton X-100-1% BSA, cells were stained with 0.165 mM rhodamine-phalloidin for 30 minutes. The amount of F-actin was measured in a FACSAria flow cytometer (Becton Dickinson, Franklin Lakes, NJ, USA) at 554 nm excitation and 573 nm emission. At least 1000 events per sample were analyzed. Data analysis was performed using the Summit software version 4.3 (Dako Colorado Inc, USA); the results expressed as the mean of fluorescence intensity (rhodamine-phalloidin in Buserelin-stimulated samples/rhodamine-phalloidin in control samples).

### Confocal microscopy of F-actin

Arrangement of F-actin in transfected cells was visualized by confocal microscopy as described elsewhere [36]. Cells cultured on Histogrip (Invitrogen)-coated coverslips were incubated in serum-free medium with Buserelin (10^-7^M) during 24 hours. F-actin was stained as described above and mounted on slides cover with ProLong solution (Invitrogen). Samples were then visualized in a Leica TCS SP5 MP multiphoton microscope (Leica Microsystems,Wetzlar, Germany).

Focal adhesion (FA) and F-actin arrangement in adherent cells to Collagen I were also evaluated by confocal microscopy. Collagen I (Sigma)-coated coverslips were placed in 24-well plates and transfected. Twenty-four hours after transfection, cells (80,000/well) were stimulated with Buserelin and stained with rhodamine-phalloidin as described above. Mouse anti-vinculin IgG monoclonal antibody (at a 1:200 dilution in PBS) and FITC-conjugated anti-mouse IgG antibody (Millipore, Temecula CA, USA) were added in tandem to visualize focal adhesion [39]. Samples were mounted and visualized as described above.

### Measurement of Rho activity

Cells were plated in Collagen I-precoated, 10 mm culture dishes (at a density of 2.125 x 10^6^ cells/dish), transfected and exposed to 10^-7^M of Buserelin in Lebovitz’s medium for 24 hours. Measurement of GnRHa-stimulated active RhoA-GTP was performed by a pull-down assay employing the Rho-binding domain (RBD) of Rhotekin coupled to glutathione-S-transferase-sepharose (GST) (GE Healthcare Bio-Science, Uppsala, Sweden) and subsequent immunoblotting. RhoA-GTP was eluted with Laemmli buffer following the protocol described previously with minor modifications [40]. Eluates were electrophoresed in 7.5% SDS-PAGE and transferred to polyvinylidene fluoride membranes (Millipore), and blots were probed with mouse anti-Rho monoclonal antibody (Millipore) at a 1:1000 dilution. RhoA-GTP and total RhoA (from no pull-down control extracts) levels were measured by densitometry. Results are expressed as the ratio of RBD/GST-bound Rho (RhoA-GTP)/total RhoA levels.

### Adhesion assays to Collagen I

Cell adhesion to Collagen I was determined by a colorimetric assay [41]. Transfected cells (20,000/well) cultured in Collagen I-coated 96-well plates were incubated for 24 hours at 37°C in FCS-free medium in presence or absence of Buserelin (10^-7^M). Adherent cells were fixed and stained with 0.1% crystal violet (Sigma) in methanol. The absorbance of sodium citrate (0.1 M)-extracted dye was then measured at 595 nm.

### Quantification of F-actin during cell adhesion and cell invasion

The amount of F-actin present in transfected, Collagen I-adherent cells incubated in the presence or absence of Buserelin (10^-7^M) as well as that present during of invasiveness conditions (i.e. cells cultured in the presence of FCS (10%)) were measured by fluorometry following the protocol described above [42].

### Invasion assays

Invasion assays were carried out in 6.5 mm, Collagen I (10 mg/ml)-coated Transwell Chambers separated by a semipermeable membrane with a 8-μm pore size (Costar) [43]. Cells were transfected as described above, detached from culture plates and resuspended in serum-free Leibovitz’s medium containing 0.1% BSA. One hundred thousand cells were added to the upper chamber and then incubated in the presence or absence of 10^-7^M of Buserelin. Cells were allowed to migrate to the lower chamber (containing Leibovitz’s medium/10% FCS, with or without GnRHa) during 24 h at 37°C in 5% CO_2_, and migrated cells were collected, pelleted, resuspended in PBS, and counted [44].

### Statistical analysis

All experiments were performed in triplicate incubations. Data were analyzed by one-way analysis of variance (ANOVA) followed by the Tukey’s multiple comparison test. A value of *P*<*0*.*05* was considered statistically significant. Statistical tests were performed using the GraphPad software (GraphPad Software Inc., v. 4.1, La Jolla, CA, USA).

## Results

### Expression and functionality of transfected GnRHRs in MDA-MB-231 breast cancer cells

**Figure 1:**
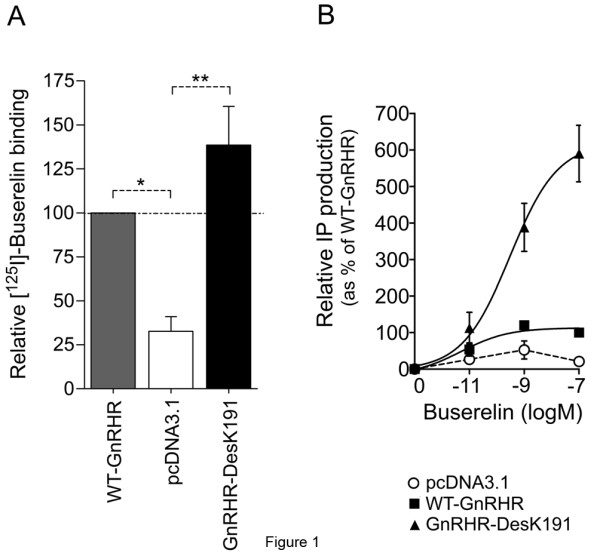
**[**^**125**^**I]-Buserelin binding and IP production in MDA cells expressing the WT GnRHR and GnRHR-DesK191 mutant. A**. Specific [^125^I]-Buserelin binding to MDA cells transfected with the empty vector (pcDNA3.1), the WT GnRHR and the GnRHR-DesK191 cDNA constructs. **B**. Inositol phosphate dose–response curves for Buserelin in MDA cells transiently expressing the WT GnRHR and GnRHR-DesK191 mutant. Maximal IP production in cells transfected with the WT GnRHR cDNA was set as 100% and all other values are expressed relative to this. Assays were performed in triplicate incubations and the results shown are the means ± SEM from three independent experiments. *p<0.001; ** p<0.01 vs pcDNA3.1.

Specific binding sites for [^125^I]-Buserelin and IP production in response to agonist exposure were detected in WT GnRHR-transfected cells. Since plasma cell surface expression of the transfected WT GnRHR was relatively low in this tumor cell line, the over-expressed mutant form, GnRHR-DesK191 was also employed to explore the effect of increased cell surface membrane-expressed receptor levels on several cell markers associated with locomotion dynamics. Compared to the WT GnRHR, specific [^125^I]-Buserelin binding and Buserelin-stimulated IP production of cells transfected with the GnRHR-DesK191 were considerably increased (to 139 ± 19% and 590 ± 134% of WT levels, for total binding and IP production, respectively) (Figures 1A and B). Relative [^125^I]-Buserelin binding affinities were similar for the endogenous receptor (empty vector-transfected) (*K*_*i*_, 1.03 ± 0.20 μM), and the WT (*K*_*i*_, 0.50 ± 0.15 μM) and DesK191 (*K*_*i*_, 0.70 ± 0.20μM) transfected GnRHRs, as disclosed by radioligand-binding assays. Nevertheless, the IP response was considerably reduced (by ~80%) in cells transfected with the empty vector, thus reflecting the low levels of endogenously expressed GnRHR in MDA-MB-231 breast cancer cells (Figures 1A and B).

### Effect of buserelin-stimulated GnRHR in actin polymerization

**Figure 2:**
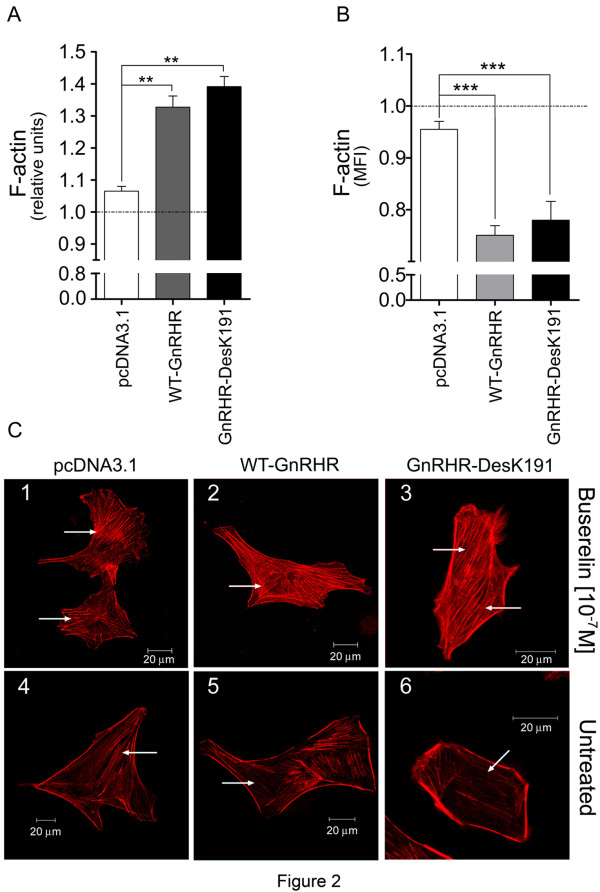
**Effects of Buserelin on actin polmerization in MDA cells. A**. Buserelin (10^-7^M)-stimulated relative F-actin levels (in arbitrary units) in adherent MDA cells transfected with the empty vector, the WT GnRHR or the GnRHR-DesK191 cDNAs, as determined by fluorometry. Cells expressing the WT and DesK191 GnRHRs exhibited a significant increase in polymerized actin in response to the GnRHa. In each group (cells transfected with the empty vector, WT GnRHR or GnRHR-DesK191), basal F-actin (i.e. no treatment) was set to 1.0 and Buserelin-stimulated levels were expressed relative to this (horizontal line). **B**. Relative F-actin levels measured by flow cytometry in suspended MDA cells transfected with the empty vector, the WT GnRHR or the GnRHR-DesK191 cDNA constructs. A significant decrease in polymerized actin was observed in response to the GnRHa. **C**. Representative images from confocal microscopy of Buserelin (10^-7^M)-stimulated MDA cells transfected with the empty vector, the WT GnRHR or the GnRHR-DesK191 cDNAs. Compared with untreated cells (lower panel) an increase in stress fibers (arrows) was apparent in WT GnRHR and GnRHR-DesK191 cells exposed to the GnRHa (upper panel). Bar: 20μm. The results shown in A and B are the means ± SEM from 3 independent experiments. ** p< 0.05; *** p<0.05.

The effect of GnRHR in F-actin cytoskeleton remodeling was assessed in adherent MDA cells exposed to a saturating concentration of Buserelin (10^-7^M). F-actin was stained with rhodamine-phalloidin and quantified for fluorometry. A significant (p< 0.05) increase in the relative amount of F-actin in WT and DesK191 GnRHRs-expressing cells was observed in response to Buserelin (Figure 2A). Although the amount of F-actin was higher in cells transfected with the GnRHR-DesK191 than in those transfected with the WT receptor, the difference did not reach statistical significance. Conversely, actin polymerization promoted by Buserelin-stimulated GnRHR and GnRHR-DesK191 was significantly decreased in non-adherent (p<0.05; Figure 2B). Thus, GnRH promoted actin polymerization only in adherent cells.

### Actin cytoskeleton arrangement after GnRHR activation in MDA cells

Although the images did not show any substantial change in the morphology of cells expressing the WT and DesK191 GnRHRs in response to a saturating concentration of Buserelin (Figures 2C 1 and 4), a remarkable increase in stress fibers crossing the cell body was observed as a result of GnRHa exposure (Figures 2C 2–3 and 5–6).

### Rho activity in MDA cell adhered to Collagen I

**Figure 3:**
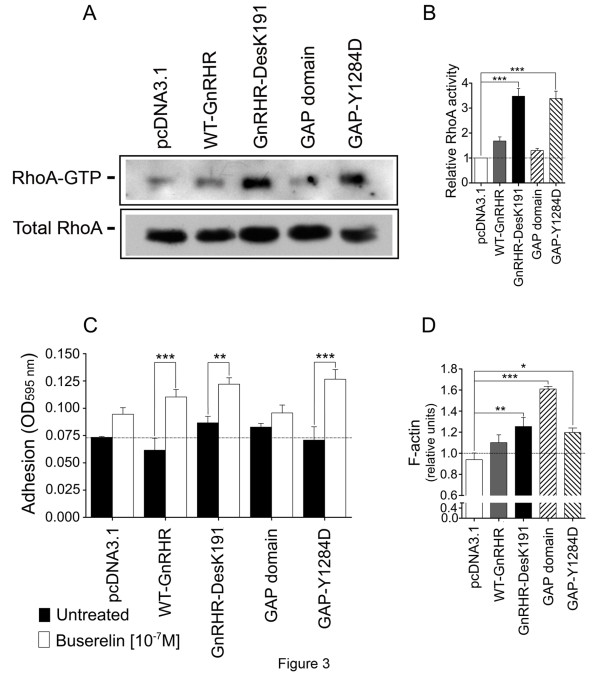
**Effects of Buserelin on RhoA GTP expression and cell adhesion to Collagen I in MDA cells. A**. Representative autoradiogram from Western blots showing the effects of Buserelin on RhoA expression in MDA cells transfected with the empty vector, the WT GnRHR and the GnRH-DesK191 cDNAs (lanes 1 to 3) or co-transfected with the WT GnRHR and p190RhoGAP or GAP-Y1284D cDNAs (lanes 4 and 5, respectively). **B**. Densitometric analysis of Rho GTP activity as determined by pull-down assays and Western blotting of extracts from cells transfected or co-transfected with the different expression plasmids and exposed to 10^-7^M Buserelin or vehicle. **C**. Assessment of adhesion to Collagen I in cells transfected or co-transfected with the different GnRHRs and GAP expression plasmids described in A and exposed to Buserelin or vehicle. **D**. Relative F-actin levels in the cell adhesion experiments shown in C, as disclosed by fluorometry. The results shown in B, C, and D are the means ± SEM from 3 independent experiments. *p<0.05; **P < 0.01; ***p< 0.001.

The increase of F-actin as stress fibers in cells exposed to Buserelin, strongly suggested that the GnRH/GnRHR system might be linked to activation of RhoA GTPase, which is responsible of stress fiber formation and focal adhesion assembly [8]. To determine the impact GnRHR activation on RhoA response, the levels of GTP-loading RhoA were analyzed in MDA cells adhered to Collagen I and stimulated with Buserelin. Negligible levels of GTP-RhoA were observed in cells transfected with the empty pcDNA3.1 vector, even in the presence of saturating concentrations of Buserelin (Figure 3A). In contrast, GTP-RhoA levels were significantly (p<0.01) increased in GnRHa-stimulated WT GnRHR- and GnRHR-DesK191-transfected cells, indicating that RhoA was activated by GnRH in MDA cells. In order to more deeply explore the association between RhoA and GnRHR activation in these cells, the GAP domain of p190RhoGAP as well as the dominant negative form of this domain (GAP-Y1284D) were co-transfected with the GnRHR and the Rho-GTP levels were determined after Buserelin stimulation. Under these conditions, GTP-RhoA protein levels were either suppressed or unaffected in cells transfected with the GAP domain or the GAP-Y1284D, respectively (Figure 3A and B).

### Attachment to Collagen I and quantification of F-actin in MDA cells

Cell attachment to the ECM is a function linked to RhoA and actin cytoskeleton dynamics [45]. Keeping this in mind, the effects of GnRHR activation on Collagen I adhesion and actin polymerization during this condition were determined. A substantial increase in cell adhesion following GnRHa stimulation was observed in WT GnRHR-, GnRHR-DesK191-transfected, and GAP-Y1284D/GnRHR-co-transfected cells. By contrast, Buserelin-stimulated adhesion was completely abolished in GAP domain/GnRHR-co-transfected cells (Figure 3C). As expected, the amount of F-actin in cell adhesion conditions was increased (p<0.01) after Buserelin activation in GnRHR-, GnRHR-DesK191- and GAP-Y1284D-transfected cells. Interesting, although in cells transfected with the GAP domain the amount of F-actin was the highest, polymerized actin was observed only in the periphery but not across the cell body (see below).

### Arrangement of focal adhesion and F-actin upon GnRHR activation in MDA cells

**Figure 4:**
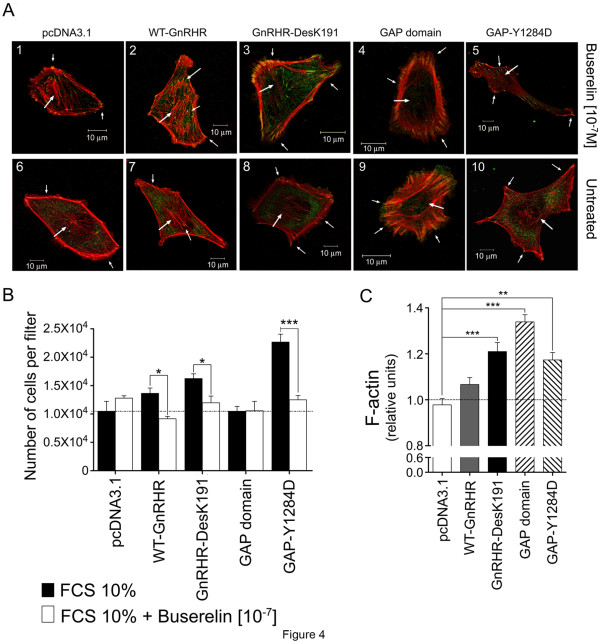
**Effects of Buserelin on focal adhesion assembly and Collagen I invasion in MDA cells. A**. Focal adhesion and F-actin arrangement as disclosed by confocal microscopy. MDA cells were transfected with the WT and DesK191 GnRHRs or co-transfected the WT GnRHR and GAP variants cDNAs, and exposed to 10^-7^M of Buserelin (upper panel) or vehicle (lower panel). The cells were then fixed and F-actin and Vinculin were stained as described in Materials and Methods. An increase in focal adhesion and stress fibers can be observed in cells overexpressing the GnRHRs as well as in those overexpressing the WT GnRHR and GAP-Y1284D (arrows in panels 2, 3 and 5), whereas cells co-transfected with the WT GnRHR and the GAP domain cDNAs exhibited decreased focal adhesion and marked accumulation of F-actin in the periphery (arrows in panels 4 and 9). Similar results were found in two other experiments. Bar: 10μm. **B**. The effect of GnRHR activation by Buserelin on cell invasion capacity as determined by invasion assays in Collagen-1-covered Transwell Chambers. **C**. F-actin levels in cell invasion. Transfected cells were attached to Collagen and exposed to Buserelin (10^-7^M) or vehicle in 10% FCS-supplemented medium, and the amount of F-actin was determined by fluorometry. The results shown in B and C are the means ± SEM from 3 independent experiments. *p<0.05; **p< 0.01; ***p< 0.001.

Cell attachment takes place through formation of focal adhesion complexes via RhoA activity [46]. These adhesion complexes favor the interactions between ECM-linked integrins and the actin cytoskeleton, as well as with a number of other cytoplasmic proteins, including talin, vinculin, paxillin, and alpha-actinin [47]. Since formation of focal adhesion reflects cell adhesion to the ECM, identification of these structures by vinculin immunostaining was conducted in transfected MDA cells plated on Collagen I. In agonist-stimulated GnRHR, GnRHR-DesK191 and GAP-Y1284D-transfected cells, accumulation of intense fluorescent dots revealed the presence of FA as well as high amount of stress fibers across the cell body (Figure 4A, compare arrows in panels 2, 3 and 5 with arrows in panels 7, 8 and 10). On the other hand, treatment of GAP domain-transfected cells led to complete absence of fluorescent vinculin dots and abolishment of stress fibers formation, indicating absence of FA in these particular cells. As noted above, high amounts of peripheral F-actin were detected in GAP-domain-transfected cells (Figure 4A, compare arrows in panels 4 and 9 with arrows in panels 1 and 6).

### Effects of GnRHR activation on invasion to Collagen I and measurement of F-actin in invasion conditions

Since RhoA plays a pivotal role in cell migration through regulating cytoskeletal changes and matrix adhesion dynamics [46], invasion of MDA cells transfected with the GnRHRs to Collagen I was evaluated. In Transwells Chambers covered with Collagen I and stimulated with Buserelin, GnRHR-, GnRHR-DesK191, and GAP-Y1284D-transfected cells showed a substantial reduction in invasion ability (Figure 4B). This inhibition was abrogated in the absence of active GTP-RhoA (GAP-domain-transfected cells) (Figure 4B). Measurement of polymerized actin during invasion showed that in contrast to control, empty vector-transfected cells, GnRHR and mainly GnRHR-DesK191 and GAP-Y1284D-transfected cells exhibited a marked increase in the amount of F-actin in the presence of Buserelin (Figure 4C), a finding that correlated with their ability to adhere to Collagen I under similar conditions (Figure 4B). Analogously with the adhesion experiments (see above), the amount of F-actin detected in invasion assays was the highest in GAP domain-transfected cells (Figure 4C).

## Discussion

Metastases at distant sites are the main cause of death in patients with breast cancer [48]. The metastatic process involves a series of events in which changes in cell motility represent the hallmark of invasion and the initial step in metastasis [6]. Over the past years, it has been clearly established that GnRH and its receptor are expressed in many extra-pituitary tissues and malignant tumors from the reproductive system, including the breast [26,27]. It has also been shown that binding of GnRH to breast malignant tumor cells results in growth modulation [18] and inhibition of metastatic capacity [20]. Although activation of some signaling pathways and effectors proteins involved in GnRHR-regulated cell motility have been reported [17,24], the molecular mechanism(s) whereby the GnRH/GnRHR system suppresses cell migration is still unclear.

In the present study we assessed the effect of GnRH on the invasiveness capacity of human breast cancer MDA-MB-231 cells, an aggressive, highly invasive, and estrogen unresponsive cell line [49]. To this end, we overexpressed the GnRHR in MDA cells and analyzed the effects of its cognate ligand on the pathways leading to actin cytoskeleton activation and cell adhesion. MDA cells transfected with the GnRHR (WT and DesK191) cDNAs, specifically bound ^125^I]-Buserelin and produced higher levels of the second messenger IP in response to the GnRH analog than untransfected cells, overcoming the problem related to the low naturally expressed GnRHR levels in breast cancer cell lines [50,51]. In fact, the increased expression levels and IP response to GnRHa detected in GnRHR-transfected MDA cells, emulated those previously detected in breast cancer cells exhibiting high GnRHR expression levels [52]. Here we confirm that to detect relevant effects of GnRH on breast cancer cells function, it is necessary to substantially increase cell surface plasma membrane receptor levels, which is an important issue given that the number of GnRHRs is highly variable in malignant breast tumors tissue [51]. In this scenario, measurement of GnRHR density in malignant breast tissue may be useful as a surrogate marker to predict the tumor responsiveness to GnRHa administration.

We have shown that in MDA cells, GnRHRs were able to promote IP production upon activation by agonist. Although we did not detect measurable changes in cAMP levels after exposure to GnRHa in this particular cell line (not shown), previous studies have found that the inhibitory effects of GnRHa on other reproductive cancers (including prostate and endometrial cancer) is mediated by the G_αi_ protein [53-55]. Our data are consistent with previous studies in MCF-7 breast cancer cells, in which the GnRH/GnRHR system was capable to selectively promote IP production [52]. These data support the idea that in extrapituitary tissues, the GnRHR may couple to different G proteins and activate distinct signaling pathways depending on the cell context, the particular GnRH analog employed to activate the receptor, and also probably the receptor density [56,57].

Actin polymerization is involved in cell migration and thus is important in determining the invasiveness ability of cancer cells [56]. Our results showed that Buserelin promoted actin polymerization as stress fibers in WT and Desk191 GnRHR-transfected adherent MDA cells, thus suggesting that activation of the GnRH/GnRHR system may be involved in the migratory potential of these malignant cells. Since Buserelin-stimulated MDA cells displayed many stress fibers and high F-actin levels, we analyzed the effects of GnRHa on RhoA, a small GTPase involved in actin polymerization and formation of stress fibers. In fact, previous studies have shown the effect of GnRH on actin cytoskeleton via other members of the Rho GTPases family [22,23]. We found that in Collagen I-adherent MDA cells, exposure to GnRHa increased RhoA-GTP levels and paralleled the amount of stress fibers. The effects of GnRH in RhoA-GTP were verified by co-transfection assays employing the GAP domain of p190RhoGAP and its dominant negative mutant, GAP-Y1284D [33]. p190RhoGAP is a specific GAP for RhoA and its effect represents more than 60% of the overall GAP activity in the cell [57,58]. The results showed that GTPase RhoA levels were abolished or unaltered in the presence of the GAP domain or GAP-Y1284D, respectively, thus indicating the specificity of the GnRH/GnRHR system on this particular small G protein. Concurrently, these data indicates that the effects of GnRH on actin polymerization and stress fibers assembly are mediated through activation of RhoA in Collagen I-attached MDA cells.

To demonstrate that GnRH-activated GTP RhoA promotes cell adhesion and thus may represent one of the mechanisms whereby this G protein inhibits cell migration, cell adhesion assays as well as confocal visualization of FA (substrate binding sites) were performed. In fact, previous studies have shown that RhoA activity supports efficient substrate adhesion, reduces cell detachment rate, and attenuates cell locomotion [59-61]. Our results showed that exposure of Collagen-I-adherent MDA cells to the GnRHa promoted cell adhesion to substrate and increased the number of FA. Further, cell invisiveness of these GnRHa-exposed cells was abolished as disclosed by invasion assays in Collagen-I-covered Transwell Chambers.

Actin polymerization leads to membrane protrusion and extracellular cell-matrix adhesion, which are generally considered as markers of the migration capacity of different cell types [62]. In this vein, it was interesting to find that in MDA cells co-transfected with the GnRHR and the GAP domain, stimulation with Buserelin did not promote detectable increments in cell adhesion to substrate - but paradoxically, it increased the levels of F-actin at the periphery of the cells. The observation that GAP domain-cotransfected cells additionally showed membrane protrusions similar to lamellipodia, suggests that continuous activation of other GTPases, such as Rac1, was present in these cells. In fact, previous studies have demonstrated the ability of GnRH to stimulate this particular GTPase [22].

Our findings in MDA cells exposed to GnRH has also been observed in other cell lines, and apparently the effects of this decapeptide on cell migration depend on the cell context. For example, it has been shown that GnRH-mediated attenuation in migration capacity of DU145 cells (prostate cancer-derived) is associated with an increase in the amount of stress fibers and with RhoA activation as well. By contrast, in TSU-Pr1 cells (also derived from prostate cancer cells) GnRH favors cell migration through mechanisms mediated by the GTPasas Rac1 and Cdc42, and by formation of filipodia and lamellipodia [23]. Our results suggests that in MDA cells transfected with the GAP domain, the absence of active RhoA GTPase promoted loss of the FA and hence in their ability to adhere to substrate as it was observed in response to GnRHa. In this scenario, the loss of migratory capacity of these cells might have resulted from the relative decrease in RhoA GTPase levels, since it is well known that cell invasion requires the concourse of several small G proteins [62].

## Conclusion

In the present study, we provide evidence demonstrating that in the highly invasive human breast cancer MDA-MB-231 cell line, activation of the GnRHR promotes RhoA activation, actin cytoskeleton remodeling and a remarkable increase in cell adhesion to substrate. Concurrently, these data may explain the ability of GnRH to reduce the metastatic potential and invasiveness of malignant breast tumor cells.

## Competing interests

The authors declare no competing interests.

## Authors’ contributions

AAR and MHR participated in the study design and the experimental studies. GMN performed the radioligand binding assays and IP production experiments. JV made the GAP domain and GAP-Y1284D DNA constructs. FAV performed the statistical analysis and AAR, AUA, and PMC. participated in the interpretation of the results and preparation of the manuscript. All authors read and approved the final manuscript.

## Pre-publication history

The pre-publication history for this paper can be accessed here:

http://www.biomedcentral.com/1471-2407/12/550/prepub
